# Modelling self-management pathways for people with diabetes in primary care

**DOI:** 10.1186/s12875-015-0325-7

**Published:** 2015-09-02

**Authors:** Marion L. Penn, Anne P. Kennedy, Ivaylo I. Vassilev, Carolyn A. Chew-Graham, Joanne Protheroe, Anne Rogers, Tom Monks

**Affiliations:** Southampton General Hospital, Mailpoint 11, AA72, South Academic Block, Tremona Road, Southampton, SO16 6YD UK; NIHR Collaboration for Leadership in Applied Health Research (CLAHRC) Wessex, Faculty of Health Sciences, University of Southampton, Highfield, Southampton, SO17 1BJ UK; Research Institute, Primary Care & Health Sciences, and NIHR Collaboration for Leadership in Applied Health Research (CLAHRC) West Midlands, Keele University, Keele, Staffordshire, ST5 5BG UK

## Abstract

**Background:**

Self-management support to facilitate people with type 2 diabetes to effectively manage their condition is complex to implement. Organisational and system elements operating in relation to providing optimal self-management support in primary care are poorly understood. We have applied operational research techniques to model pathways in primary care to explore and illuminate the processes and points where people struggle to find self-management support.

**Methods:**

Primary care clinicians and support staff in 21 NHS general practices created maps to represent their experience of patients’ progress through the system following diagnosis. These were collated into a combined pathway. Following consideration of how patients reduce dependency on the system to become enhanced self-managers, a model was created to show the influences on patients’ pathways to self-management.

**Results:**

Following establishment of diagnosis and treatment, appointment frequency decreases and patient self-management is expected to increase. However, capacity to consistently assess self-management capabilities; provide self-management support; or enhance patient-led self-care activities is missing from the pathways. Appointment frequencies are orientated to bio-medical monitoring rather than increasing the ability to mobilise resources or undertake self-management activities.

**Conclusions:**

The model provides a clear visual picture of the complexities implicated in achieving optimal self-management support. Self-management is quickly hidden from view in a system orientated to treatment delivery rather than to enhancing patient self-management. The model created highlights the limited self-management support currently provided and illuminates points where service change might impact on providing support for self-management. Ensuring professionals are aware of locally available support and people’s existing network support has potential to provide appropriate and timely direction to community facilities and the mobilisation of resources.

## Background

Self-management actions by individuals with long-term conditions and the support they receive from primary care practitioners has been viewed as an essential element of managing and optimising positive health outcomes for long-term conditions (LTCs) generally and for type 2 diabetes in particular [1–3]. For individuals to self-manage diabetes, a number of key items have been identified. In addition to diet, activity, medication regulation and self-monitoring, a sense of well-being dependent on a feeling of control of the diabetes is relevant [4]. Beyond the individual, social network mechanisms of support [5], social and political conditions and contact with services impact on how diabetes is experienced and managed [6]. Table 1 lists the UK National Institute for Health and Care Excellence (NICE) quality statements for diabetes management and indicates how these are optimally translated into self-management support within primary care, secondary and specialist care and the wider community.

**Table 1:** UK diabetes quality statements and translation into self-management support

Diabetes Quality statements NICE 2011 (revised 2012)	How these translate into primary care self-management support or referral outside primary care
https://www.nice.org.uk/guidance/qs6/chapter/List-of-statements	
Statement 1. People with diabetes and/or their carers receive a structured educational programme that fulfils the nationally agreed criteria from the time of diagnosis, with annual review and access to ongoing education.	• Referred to education programme commissioned by local Clinical Commissioning Group which takes place in the community
	• Attendance checked at annual review with Practice Nurse(PN)/Health Care Assistant
Statement 2. People with diabetes receive personalised advice on nutrition and physical activity from an appropriately trained healthcare professional or as part of a structured educational programme.	• Provided by PN; or
	• Referred to specialist dietician; or
	• Covered in education programme
Statement 3. People with diabetes participate in annual care planning which leads to documented agreed goals and an action plan.	• Covered during regular and annual reviews within the practice
Statement 4. People with diabetes agree with their healthcare professional a documented personalised HbA1c target, usually between 48 mmol/mol and 58 mmol/mol (6.5 % and 7.5 %), and receive an ongoing review of treatment to minimise hypoglycaemia.	• Covered during regular and annual reviews within the practice
Statement 5. People with diabetes agree with their healthcare professional to start, review and stop medications to lower blood glucose, blood pressure and blood lipids in accordance with NICE guidance.	• Covered during regular and annual reviews within the practice
Statement 6. Trained healthcare professionals initiate and manage therapy with insulin [where indicated] within a structured programme that includes dose titration by the person with diabetes.	• Covered in education programme; or
	• Takes place during regular and annual reviews within the practice where PN has been trained
Statement 7. Women of childbearing age with diabetes are regularly informed of the benefits of preconception glycaemic control and of any risks, including medication, that may harm an unborn child. Women with diabetes planning a pregnancy are offered preconception advice, and those not planning a pregnancy are offered advice on contraception.	• Takes place during regular and annual reviews within the practice
	• Referred to specialised antenatal care
Statement 8. People with diabetes receive an annual assessment for the risk and presence of the complications of diabetes, and these are managed appropriately.	• Takes place during annual review within the practice
Statement 9. People with diabetes are assessed for psychological problems, which are then managed appropriately.	• Takes place during regular and annual reviews within the practice
	• Referred to appropriate mental health team
Statement 10. People with diabetes at risk of foot ulceration receive regular review by a foot protection team in accordance with NICE guidance.	• Provided by foot protection team/podiatrist following referral from primary care
Statement 11. People with diabetes with a foot problem requiring urgent medical attention are referred to and treated by a multidisciplinary foot care team within 24 h.	• Provided by foot protection team/podiatrist following referral from primary care
Statement 12. People with diabetes admitted to hospital are cared for by appropriately trained staff, provided with access to a specialist diabetes team, and given the choice of self-monitoring and managing their own insulin.	• Specialist diabetes team in secondary care
Statement 13. People admitted to hospital with diabetic ketoacidosis receive educational and psychological support prior to discharge and are followed up by a specialist diabetes team.	• Specialist diabetes team in secondary care
Statement 14. People with diabetes who have experienced hypoglycaemia requiring medical attention are referred to a specialist diabetes team.	• Specialist diabetes team in secondary care

In addition – People with diabetes need to have annual screening at accredited optician or diabetes screening service

In countries with advanced systems of welfare and health care provision, there has been a comprehensive re-structuring of LTC management at the interface between primary and secondary care. In the UK context, care for people with type 2 diabetes has moved from hospitals to primary care and this has been found to influence the perception of the condition with potential consequences for how seriously people take advice on lifestyle changes [7, 8]. The latter constitutes the recent context from within which the delivery of support for type 2 diabetes self-management in the UK has been undertaken [1, 3, 9]. Across Europe, self-management support has been found to be varied and far from adequate [10, 11]. Advantages of relocation to primary care for patients has been identified as including convenient, local care, which reduces the financial and time burdens of going to the hospital and thus makes life easier [12]. Research focusing on experiences with primary healthcare after the re-structuring (particularly where diagnoses are established in primary healthcare) found that people report the perception of a lower frequency of contact with staff ‘specialised’ in diabetes care and a questioning amongst some of adequacy of competence and knowledge about diabetes held by primary care practitioners [12, 13]. Previous research suggests that, to some extent, the micro-level individual experiences and the ability to self-manage adequately is bound up with the structure, organisation and delivery of primary care practice [14].

In response to the rising prevalence of type 2 diabetes and the cost to health systems [15–17], a plethora of national and international guidelines and standards related to self-management support for people with diabetes have been developed. Guidelines (see Table 1) point to the requirement for structured and tailored patient education which includes: lifestyle management advice (nutrition and exercise), medication use, monitoring blood glucose, detecting and managing complications (eye disease, kidney disease, nerve damage and depression) [18, 19].

Here we set out to model and critically appraise the pathways in primary care which currently operate to increase the ability of people with diabetes to self-manage. In the United Kingdom National Health Service (NHS) system, primary care is now where most people with type 2 diabetes are managed. In the UK, diabetes was one of the conditions included in the original Quality and Outcomes Framework (QOF) pay-for-performance scheme for primary care introduced in 2004, and modified annually thereafter. Fifteen of 101 quality indicators in the scheme currently (March 2015) relate to diabetes care, [20].

Everyone with a LTC manages their condition to some degree and this is particularly salient and important in relation to diabetes. However, it is recognised that this is not something an individual can achieve successfully on their own or exclusively with a health care professional although the underlying assumptions are that General Practitioners (GPs) and Practice-based Nurses (PNs) are best placed to provide support to help people make lifestyle changes and take more control over the day-to-day management of their condition (see Table 2). As Table 2 indicates, most support for successfully living with a long-term condition lies outside the NHS. Thus a clearer explanation of the role of primary care organisations in helping people access the most appropriate support is needed.

**Table 2:** Support patients can expect from the NHS

You can expect lots of support from the NHS, including:	
•	healthy lifestyle support: helping you improve your diet and exercise regime
•	information: advice about your condition and its treatment
•	training: helping you feel more confident about living with your condition
•	tools and equipment: making life easier at home
•	support networks: help with finding people to share your experiences with

http://www.nhs.uk/Planners/Yourhealth/Pages/Yourhealth.aspx

To achieve our aims, we have utilised operational research techniques to develop influence models of self-management and drawn on an exercise undertaken by staff from 30 primary care general practices. The data was obtained from primary care clinicians and support staff during a training exercise to implement a whole systems approach to improving engagement with self-management called WISE (Whole System Informing Self-management Engagement) [21]. This formed the basis of a large randomised controlled trial (RCT). The focus of WISE was to train primary care teams to provide support in an evidence-based and systematic way. In fact, WISE did not embed fully in primary care, and a process evaluation found that this was because provision of self-management support lacked relevance for clinicians and patients and did not fit with existing work [14]. These RCT findings indicate that policy presumptions of situating self-management support within primary care may be misplaced. The intention of using operational research here is to improve the understanding of why primary care is currently failing to support self-management by exploring models created by people working in primary care alongside existing knowledge about how long-term conditions are managed in the health service.

### Operational research

Operational research applies mathematical, computer and systematic modelling techniques to aid complex decision-making with the aim of improving patient outcomes and understanding of system behaviour [22]. The tools emerging from operational research and use of modelling are underutilised in health care planning and have great potential for improving service use and helping organisations to make effective change [23, 24].

Application of operational research modelling in health services research has ranged from highly quantitative work focused purely on acute needs [25], models that attempt to incorporate entire patient pathways across primary, secondary and community services [26]; to qualitative modelling where service users have taken the lead in resolving their own problem [27].

Operational research has previously been applied to aspects of the management of type 2 diabetes and includes complex data analysis to provide ongoing medical advice [28], to assist with appropriate drug selection [29], to compare the effectiveness of GP practice level interventions [23], to perform simulation modelling of retinal screening policies [30–32] and diabetic podiatry checks [33], and mathematical optimisation to assess the appropriateness and effectiveness of prevention programmes [34]. To our knowledge there are no other applications of operational research in the modelling of self-management; as such our consideration of the role of self-management for type 2 diabetes is novel.

We were interested in using operational research modelling to explore the following questions:

1. How is self-management support within primary care currently organised?
2. What is it about the current organisation of support and predominant practice that can inform our understanding as to why primary care is failing to provide effective and consistent self-management support to people with diabetes?
3. Where, how and at what point in primary care can diabetes self-management support resources be maximised?

## Methods

### Ethics

This study was approved by Salford and Trafford local research ethics committee (reference 09/H1004/6). Informed consent was obtained from the practices who took part in the training; for this part of the WISE study the ethics committee did not require individual training participants to provide consent.

### Data from the WISE training

As part of training sessions in delivering the WISE self-management support approach in primary care, GPs, nurses and admin staff were asked to create a pathway ‘from reception to self-management’ for people with diabetes. The training included an introduction to self-management support prior to the pathway exercise [35]. The purpose of this element of the training was for staff to learn about people’s roles in the practice and their impact on the way patients participate in health care with the aim of improving systems of self-management support within the practice. They were asked: “What is the route a person with diabetes takes through your practice - from appointment via reception to leaving the practice after seeing the health professional?” Depending on the practice size, they did this as a whole practice team or in smaller groups.

The health care practitioners first mapped out an ‘ideal’ path and then used different coloured post-its to identify barriers and problems to providing and promoting self-management support as well as good/optimal practice:

1. Details of how the system works
2. The ways GPs, nurses and support staff work
3. Gaps in resources for patients
4. What you consider you do well in your practice

The individual pathway maps were collected and a Microsoft Publisher Document was created for each pathway. We collected data from 21 practices for 23 diabetes pathways (two practices produced two pathways).

### Analysis

**Fig. 1:**
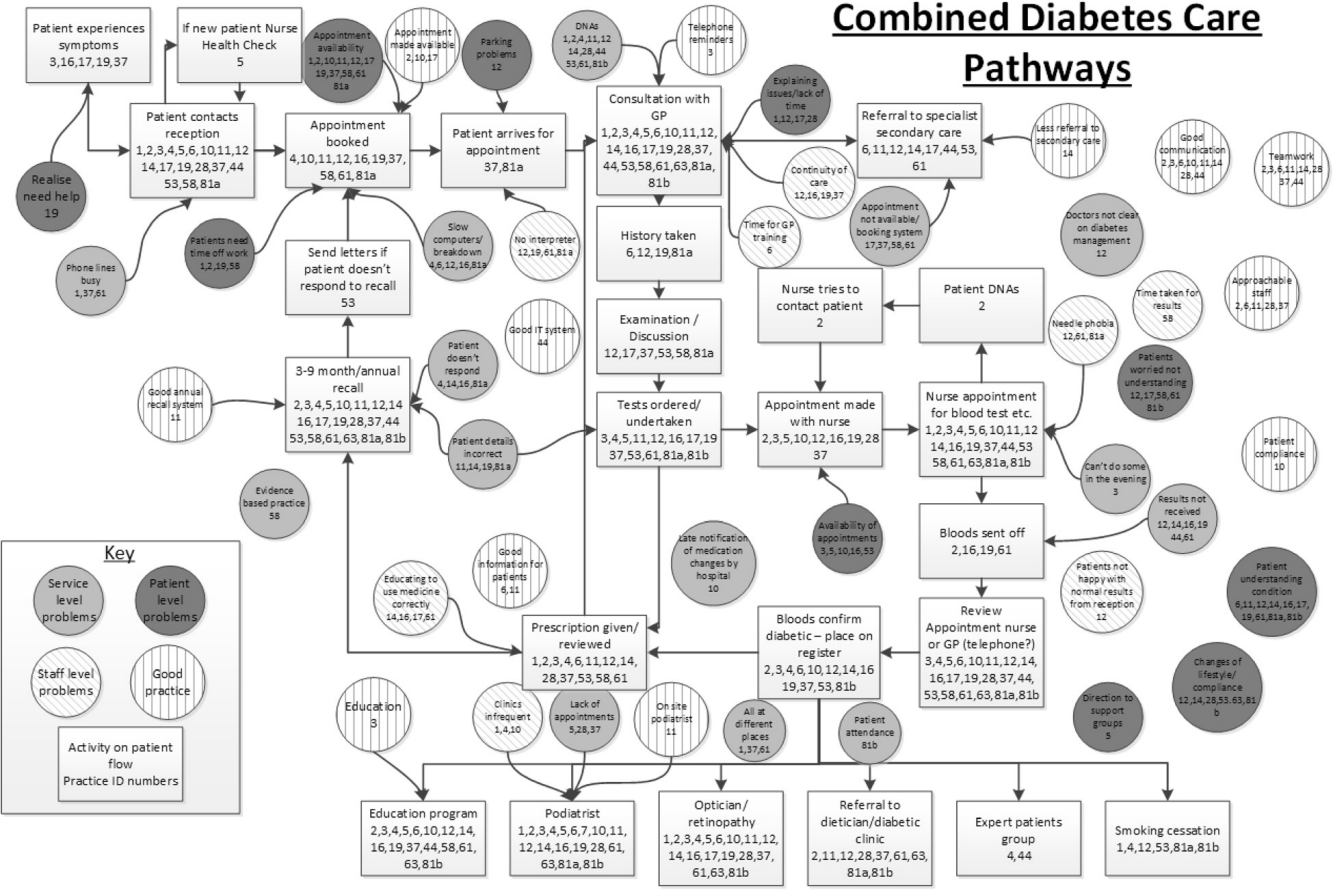
Combined GP practice pathways for diabetes patients

In order to identify common themes between the pathways and gain additional insights from comparisons between them these pathways were collated into a combined pathway. Two of the authors are experienced operational research modellers. The modellers facilitated the creation of the influence diagrams with other members of the research team (health service researchers and professionals) – and iteratively produced the model refining each time the research team met. It was this iterative process that led to the insight that the frequency of reviews is a key factor in how self-management happens. The combined pathway for diabetes is shown in Fig. 1.

In the diagram the rectangular boxes represent the stages on the flow of people with diabetes through the pathway, the numbers in each box represent the practices for which that stage appeared on the individual pathways.

The circles represent issues that clinicians perceive to affect the experience of people with diabetes; the lighter grey circles are for service level limitations; darkest grey for people with diabetes level and diagonal lines for staff level. The circles with vertical lines represent good practice that enhances the experience of people with diabetes. Again the numbers in each circle represent the practices which included those factors on their individual maps. It should be noted that for several of the issues affecting patient experience different practices had the same issue in different categories, suggesting that many issues occur at multiple levels.

Analysis led to consideration of how the frequency of reviews links to ability to self-manage and interventions to enable self-management. In the pathways activities, health professionals did not identify any self-management support practices that could be applied consistently over time after diagnosis (e.g. in the structure of the consultation or referral process). Frequency of reviews emerged as the only structured, although indirect, process through which primary care professionals engaged with people’s ability to self-manage. Specifically, there is an assumption that the self-management ability of people with diabetes would improve after diagnosis and therefore there is also an expectation that the frequency of appointments in general practice would also be reduced over time. This raises the importance of exploring the pathway of people with diabetes after diagnosis in more detail. The operational research technique used for this is influence diagrams. In the case of self-management, these models demonstrate how different service user, organisational, clinical factors and interventions either positively or negatively influence service user flow through the diabetes pathway [36, 37].

### Pathways of people with diabetes including self-management

**Fig. 2:**
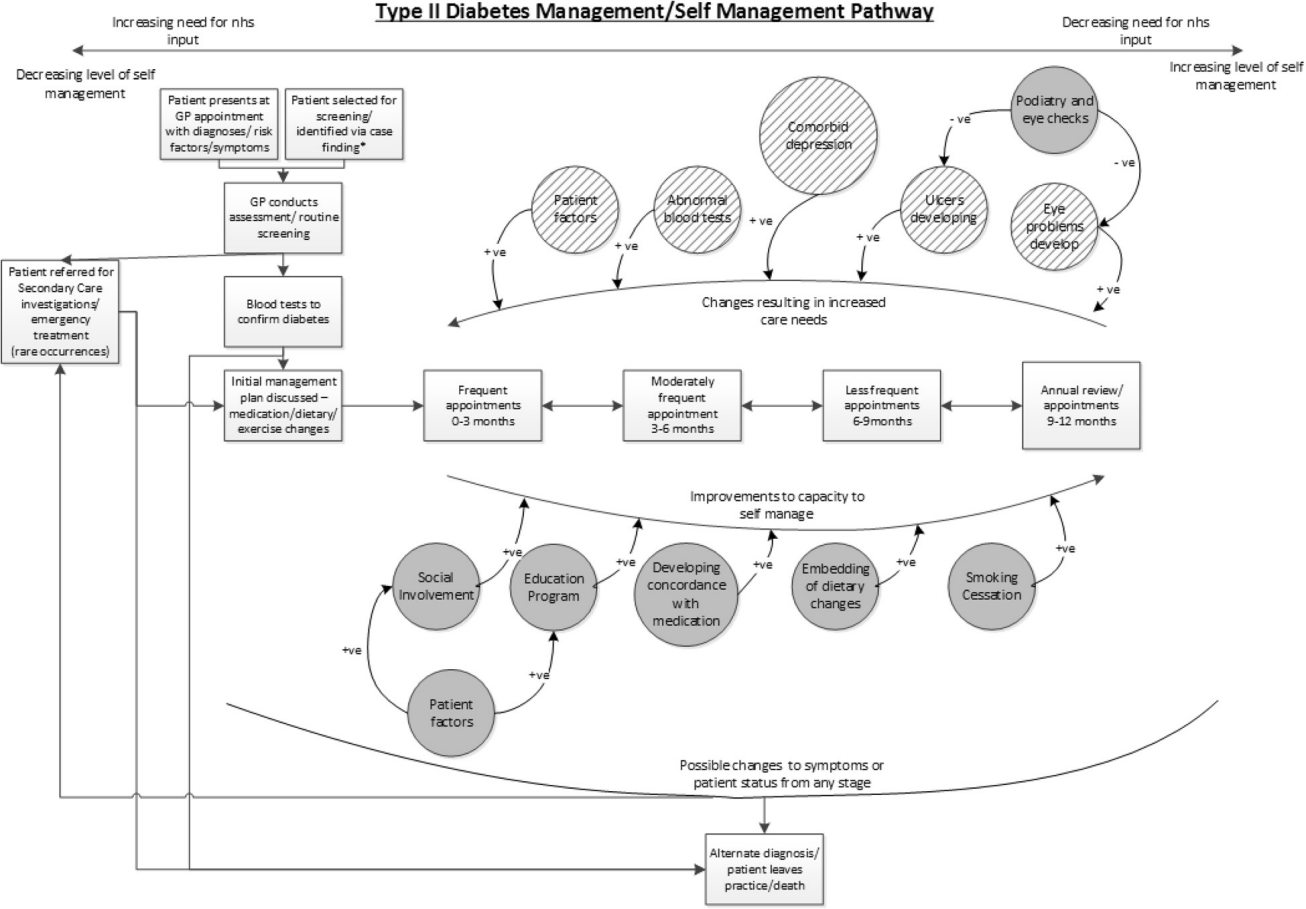
Diabetes management pathway including self-management

In addition to the information from the WISE pathways of people with diabetes; data concerning self-management support options from the WISE patient information booklets which formed part of the trial intervention [21, 38], and the NHS Choices website http://www.nhs.uk/Conditions/Diabetes/Pages/Diabetes.aspx have been drawn on for the management pathway after diagnosis. This has also been validated through clinical review with GP authors (CCG and JP) and is shown in Fig. 2.

As for Fig. 1 the rectangles represent the flow for people with diabetes. The diagram is arranged so that people with diabetes to the left of the diagram are those with the greatest need for clinical input and lowest level of self-management and those on the right are requiring less clinical input and with potentially the highest level of self-management. The items in circles are the influences affecting the flow of people with diabetes, those shaded grey are positive influences on self-management and those with diagonal lines are negative influences. The positive signs linking the influences to the flows indicate that those influences have a positive effect on the flow to which they are attached. The negative links between influences indicate that the influence at the start of the arrow has a reducing effect on the influence at the arrows point.

**Fig. 3:**
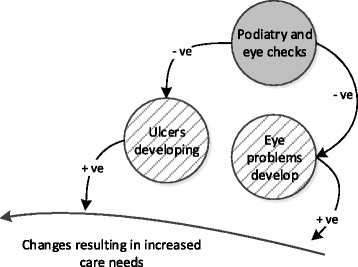
Example of how the influences are illustrated

Figure 3 is a smaller example of how the influences interact. The positive arrows from “Ulcers developing” and “Eye problems develop” to the increase in care needs indicate that either of these issues will result in an increased demand for health care. The negative arrows from “Podiatry and eye checks” to “Ulcers developing” and “Eye problems develop” indicate that the checks taking place reduces the rate at which ulcers and visual problems increase the need for treatment.

The circles relating to patient factors covers a broad range of factors, the explicit inclusion of all of which would overcomplicate the diagram making it hard to follow. These include, but are not limited to, comorbidity/multi-morbidity, health literacy, education background, deprivation and practical considerations such as transport. They can have both positive and negative effects on people with diabetes’ frequency of accessing health services.

## Results

Figure 1 illustrates the difficulties around people with diabetes’ ease of accessing healthcare particularly in making and attending appointments. Access varied between individual practices with the key differences between practices being the frequency of disease management reviews and the types of support offered to people with diabetes (e.g. not all practices referred people to education programmes, and this was not a quality indicator at the time of the WISE trial).

For all of the practices there was an emphasis on the processes surrounding initial diagnosis and management of people with type 2 diabetes. Whilst the majority of the initial practice maps mention the frequency of reviews, these were generally clustered around the review appointments made shortly after diagnosis. There were no indications of how the frequency of reviews changes over time.

There was also an absence of explicit reference to self-management in the practice maps. The problems that show up in Fig. 1 which are of most relevance to self-management are ‘patients understanding’ and ‘changes of lifestyle/compliance’, both of which are central aspects of facilitating self-management. This might indicate that while some form of self-management engagement does take place in primary care this is likely to be ad hoc and highly contingent on circumstances. For example, there was an expressed concern about systems in one practice in that ‘doctors [were] unclear about diabetes management’. An expressed lack of inter-professional knowledge about how the system works is likely to limit the confidence and capacity of practices to offer reliable and tailored self-management support.

These results coalesce with the conclusions of a process evaluation illuminating the failure of WISE to embed self-management support in primary care because of a lack of perceived relevance of self-management support for clinicians and a failure of the work of self-management support fitting with existing work demands of primary care staff [14].

Figure 2 provides a visual demonstration of a (non-exhaustive) list of factors that indicate or have a direct impact on the effectiveness of people with diabetes’ self-management, and illuminates the direction of impact that different factors including additional illnesses can have on the frequency with which people with diabetes require medical reviews (e.g. having depression could increase the frequency of reviews and increased social involvement could decrease the frequency). Figure 2 demonstrates that ideally there should be a mechanism operating around increasing people with diabetes’ self-management capabilities and reducing the level of medical input. The latter forms the premise of demand management assumptions around policies designed to increase self-management activities of a population resulting in decreased demand and utilisation of services [39].

However, while Fig. 2 identifies a wide range of factors that could potentially have an impact on self-management, it is only those that are in the upper part of the diagram that could directly trigger a change in the frequency of reviews. This is because, as illustrated by Fig. 1, the content of reviews focus on conducting bio-medical tests, assessments of illness progression, the development of complications and co-morbidities, rather than on how people with diabetes are managing overall and what support they might benefit from. This coalesces and provides more support for observational findings about: “*the underlying technical and fragmented nature of the tasks they [professionals] were required to perform had the effect of reducing opportunities for patients to offer symptoms following……. We found only two examples where the interactional environment allowed the patient to utilise this formulation to indicate a concern with their nurse”.* [40].

Additionally, pressure on the allocation of staff time is indicated around the access difficulties encountered by people with diabetes in obtaining appointments (see Fig. 1) and the time and space given over to the operationalization of specific targets measured for the QOF.

## Discussion

Previous research methods have illuminated some of the problems of implementing SMS in primary care (2, 18–20). These have tended to focus on discrete elements of primary care practice and organisation related to how professionals can make sense of a change such as the need for self-management and embed it daily practices. The creation of a model for diabetes self-management pathways in primary care provides a clearer picture of the complexities in achieving good self-management support in primary care at a systems and organisational level. In this respect the modelling provided confirmatory evidence for what has been shown in the WISE trial and process evaluation – that self-management support for long-term conditions is not well understood nor is it embedded in primary care. The added advantage here is that it provides a map of where points in the system of primary care are excluding or making the provision of support more challenging and thus of points where remedial action could be taken.

The first model (Fig. 1) highlights the barriers to accessing and taking up care for patients – many of them due to routine systems not functioning well such as problems making appointments, poor IT systems, dispersed services and lack of time. In some respects this does not differ from other patient groups [41]. Nonetheless there is evidence that the more open a system of support is the more it encourages autonomous action on the part of the patient. This is because perceived support and access into the system is relevant to people with LTCs in terms of providing background reassurance that support is available should individual self-management practices or activities fail or become too challenging [42]. In addition, there needs to be acknowledgement and expectation that support for managing long-term conditions comes from both outside and within the health system [43].

Once the work and arrangements for disease management have been established following diagnosis, the person is generally presumed to be managing well (as indicated by biological markers) and appointments become less frequent. At this point self-management becomes the norm. However, one implication of this presumption for people with diabetes is that self-management quickly becomes hidden from view which is reinforced by the systems and organisational priorities operating in primary care. The focus on the bio-medical and routine monitoring may be legacy of the move from secondary to primary care [12]. Primary care is more frequently associated with engaged patient decision-making and patient-centred support over the longer term than the more acute episodic approach characteristic of secondary care [44]. The prioritisation in primary care of a certain configuration of procedures, tests and appointments outlined in Table 1 indicate how SMS becomes invisible and does not happen within review appointments. Despite the proliferation of the notion of joint decision-making and patient participation and involvement, improvements in people with LTCs’ capacity to self-manage are not it seems overtly or visibly built into the current pathway [45]. Our study highlights that there is little opportunity for feedback within the system, for example, clinicians are not informed about attendance at education programmes unless the individual informs them, and there appeared to be no monitoring as to whether people may require information and advice to be refreshed or updated. These processes may be an unintended consequence of a system of primary care which via pay-for-performance incentivises the actions and monitoring systems of primary care professionals and de-facto disincentives those which would favour people with diabetes’ need for support around self-management [20]. The current orientation of incentivisation of specific processes of care may inadvertently detract from the central role of the individual with diabetes in operationalising, monitoring and working in partnership with primary care professionals to optimise self-management activities. Thus, the nature of patient responsibility and shared decision-making within the system is opaque. A hard-to-access health service means people with diabetes have to rely solely on support and advice during a short, and predominantly bio-medically focussed review appointment (six monthly or annually) [46]. Such monitoring appointments are not conducive to patient-centred discussions and the work needed to establish a partnership where challenges can be made by both partners concerning lifestyle change may be considered too disruptive to relationships for health care professionals to initiate and trust is hard to establish [14, 47, 48]. Regular review appointments of this orientation may also have the effect of reducing people with diabetes’ efforts to take on responsibility for self-management as they may act to devolve control for monitoring to health care professionals [49].

### Strengths

The method allows visualisation of the management of people with diabetes including factors which both positively and negatively influence their experience. Models such as ours provide a means for developing an explicit shared understanding of both the patient pathway and experience in type 2 diabetes between clinicians, healthcare managers and service users. Therefore we believe such approaches are a valuable approach to debating service change to support self-management of type 2 diabetes within the NHS.

### Limitations and further research

Our models are based on data from a single region and hence results may vary when applied elsewhere. However, given the explicit representation of the self-management pathways in our modelling approach we argue that our models serve as a clear tool to debate how one self-management pathway differs from another.

Future work will explore the feasibility of quantitative modelling of self-management pathways in type 2 diabetes, in particular using System Dynamics [50]. Such a model would mimic the current self-management pathway of people with diabetes and predict how interventions in self-management affect patient flow through the pathway which ultimately affects both healthcare costs and patient quality of life. Future modelling also needs to take into account the more open patient systems of managing using resources beyond the health system.

As the influence maps were created primarily from the WISE trial experience we did not directly include service users in their creation. Service user involvement and the design of operational research modelling is in its infancy; although some have suggested a provisional framework for involvement [51]. An area of future research with great potential is community operational research [27]. In this mode of research the role of the operational researcher and research user are reversed. Service users would develop their own methodology, often qualitative, to express their understanding and experience of a self-management pathway while the operational researcher acts a facilitator only.

A further strength is that the modelling approach employed is equally applicable to the other conditions explored in the WISE trial i.e. chronic obstructive pulmonary disease and irritable bowel syndrome. Further research could consider the commonalities between the self-management models developed.

## Conclusions

The modelling here highlights the need for a refocusing on personal tailoring of support so that it fits with eliciting expressed needs about managing activities. There are indications that such support should optimally be offered at several points during the person’s condition trajectory rather than focusing attention, as currently, mostly around the point of diagnosis. Social support networks may also be a key factor here as links to others mean experiences can be shared and learned from in a sustainable and long-term way [5]. However, there is limited awareness of local community facilities amongst primary care staff [52]. People with diabetes who are surrounded by others with personal experience of type 2 diabetes are likely to have better opportunities for mobilising support resources compared to those in networks with relatively fewer people with personal experience or knowledge. Thus for primary care professionals, increasing both their awareness of community resources and people’s existing networks of support could promote more appropriate and timely direction to support.
